# *Bulbophyllumchrysolabium* (Orchidaceae, Epidendroideae, Malaxideae), a new species from Yunnan, China

**DOI:** 10.3897/phytokeys.111.28136

**Published:** 2018-11-13

**Authors:** Lin Li, De-Ping Ye, Song-Jun Zeng

**Affiliations:** 1 Key Laboratory of Plant Resources Conservation and Sustainable Utilization, South China Botanical Garden, Chinese Academy of Sciences, Guangzhou 510650, Guangdong, China South China Botanical Garden, Chinese Academy of Sciences Guangzhou China; 2 Forest Bureau of Pu’er, Pu’er 665000, Yunnan, China Forest Bureau of Pu’er Pu'er China

**Keywords:** Menglian County, new taxa, section *Racemosae*, taxonomy

## Abstract

*Bulbophyllumchrysolabium*, a new species belonging to section Racemosae from Yunnan, China is described and illustrated. The species is related to *B.orientale* and *B.morphologorum*, but differs by having the following set of characters: obliquely broadly-based triangular petals with a long filiform apex; lip densely glandular papillose and conspicuously ciliolate along margins; lip auricles well developed, narrowly falcate, tapering to a long sharp point at the apex; stelidia subulate and twisted inwards, slightly exceeding operculum. The conservation status of *B.chrysolabium* is assessed and taxonomic notes are provided.

## Introduction

Recently, based on phylogenetic studies, all genera previously proposed in subtribe Bulbophyllinae were formally transferred to *Bulbophyllum* Thou. (Vermeulen et al. 2014). As the largest orchid genus, *Bulbophyllum* Thou. (1822: tab. 93) is pantropical and widespread, with approximately 2200 species described worldwide (Sieder et al. 2007; Vermeulen 2014). However, it is believed that there are still numerous species awaiting discovery and formal description.

The high number of species in the genus *Bulbophyllum* makes systematic study not easy due to their morphological similarities and difficulties encountered in identifying them in their vegetative states. Bulbophyllumsect.Racemosae Benth. & Hk.f. (1883: 502) includes such species. According to the revised circumscription for section Racemosae (Seidenfaden 1979, 1992; Vermeulen and O’Byrne 2011; Vermeulen 2014), members of this section are characterized by distinct or well-developed pseudobulbs bearing a single leaf; inflorescence with many densely arranged flowers in a cylindrical raceme; mobile labellum usually with proximal auricles; distinct stelidia; basal node of pedicel approximately level with floral bract attachment. During our recent botanical survey in Menglian County of south-western Yunnan, China, an unusual species was discovered and collected. After further studies, the species was confidently classified as a Bulbophyllum species of section Racemosae, with approximately 38 species mainly distributed in tropical and subtropical Asia. There are seven species recorded in China, all of which occur in Yunnan Province (Seidenfaden 1979, 1992; Tsi 1999; Li 2003; Chen and Vermeulen 2009; Ye and Li 2012; Averyanov et al. 2016). A thorough literature and herbarium specimen review indicated that it is clearly different from any other previously known taxon of the section.

## Materials and method

Morphological comparisons and assessments of *Bulbophyllumchrysolabium* and its allies in the section Racemosae were performed based on specimens from herbaria BM, E, IBSC, K, KUN, PE (acronyms according to Thiers 2018), as well as consideration of the taxonomic literature. In addition, living plants were collected and transplanted to the nursery of the South China Botanical Garden, Chinese Academy of Sciences (SCBG) for further investigation. Measurements and photographs on the fresh material were made under a stereomicroscope (Olympus MD-90). The conservation status of the putative new species was evaluated following the guidelines of the International Union for Conservation of Nature (IUCN 2017).

## Taxonomic description

### Bulbophyllum chrysolabium

Taxon classificationPlantaeAsparagalesOrchidaceae

L. Li & D.P. Ye sp. nov.

urn:lsid:ipni.org:names:77191936-1

Figures 1 , 2

#### Diagnosis.

*Bulbophyllumchrysolabium* is distinguished from all known congeners by having the following unique combination of features: obliquely broadly-based triangular petals with a long filiform apex; lip densely glandular papillose on both sides and conspicuously ciliolate along margins; lip auricles well developed, narrowly falcate, tapering to a long sharp point at the apex; stelidia subulate and twisted inwards, slightly exceeding operculum.

#### Taxonomic notes.

*Bulbophyllumchrysolabium* appears to be related to *B.orientale* Seidenf. (Seidenfaden 1979: 138), especially in narrowly falcate lip auricles and twisted stelidia, but differs in distinctly longer floral bracts (almost twice as long as the pedicel and ovary); petals with long filiform apices, a rather smaller lip (ca. 2.8 mm long), significantly glandular-papillose and ciliolate at margins; stelidia slightly exceeding operculum and distinctly longer than column. With respect to filiform petals, *B.chrysolabium* is also superficially similar to *B.morphologorum* Kräenzl. (1908: 89), however, the latter have a fat, conical protuberance or callus on the front of the column near its base and scape much longer than rachis. In addition, it has subulate, not twisted stelidia, considerably longer than operculum; lip auricles not falcate, but rather obtuse at the apex. A detailed morphological comparison between *B.chrysolabium* and its allied species is presented in Table 1.

**Table 1.** Diagnostic morphological characters distinguishing *B.chrysolabium* from its two allied species.

Character	* B. chrysolabium *	* B. morphologorum *	* B. orientale *
Floral bract	6–6.5 mm, distinctly longer than pedicel and ovary.	4.5–5 mm, distinctly longer than pedicel and ovary.	5–6 mm, nearly as long as or slightly longer than pedicel and ovary.
Scape	5–6 cm, rachis about 6.5 cm, longer than scape	10–13 cm, much longer than rachis	4–8 cm, rachis about the same length
Dorsal sepal	oblong-ovate or narrowly ovate, apex shortly aristate or apiculate, 4.5–5 mm × 1.0–1.5 mm	ovate, apex acute, 6.5–7 mm × 2.5–3 mm	broadly ovate, apex acute, ca. 7.0 × 3.5 mm,
Lateral sepals	obliquely oblong-ovate, margins strongly involute, with the apex shortly caudate, 5.5–6 mm × 2.0–2.5 mm	obliquely ovate-lanceolate, margins not involute, apex acute, 7.5–8.0 × 3.5 mm	obliquely ovate-lanceolate, margins not involute, apex acute, ca. 8.0 × 4.5 mm
Petals	apex with long filiform apices, ca. 3.0 mm long, usually contorted to spirally twisted	apex with filiform apices, ca. 1.8 mm long, not twisted	apex with cuspidate apices, ca. 0.8 mm
Lip	ca. 2.8 mm long, significantly glandular-papillose and ciliolate at margins	ca. 5 mm long, not papillate or papillose at margins	ca. 6 mm long, somewhat papillate at margins, not ciliolate
Lip auricles	narrow falcate, long acuminate with a sharp point	not falcate, rather obtuse at apex	narrow falcate, apex apiculate and unequally 2- or 3-toothed
Column callus	absent	present, conical	absent
Stelidia	subulate and twisted, exceeding operculum	subulate but not twisted, exceeding operculum	subulate and twisted, as long as or shorter than operculum

#### Type.

CHINA. Yunnan Province. Menglian Dai, Lahu and Va Autonomous County (referred to as Menglian County), 1400–1600 m alt., 23 May 2014, L. Li 1209, (holotype, IBSC! isotype, IBK!).

#### Description.

Epiphytic herbs. Rhizome rigid, creeping, 2–3 mm thick. Pseudobulbs borne on rhizome at an interval of 3–5 cm, ovoid to narrowly ovoid, 2.2–3.5 cm high, base often covered with remnant fibrous sheaths. Leaf apical, single, coriaceous, oblong to linear-oblong, 12–14 cm × 1.8–2.2 cm, subsessile, apex retuse or minutely notched. Inflorescences developing from base of pseudobulb, with pendulous densely many-flowered raceme; scape 5 to 6 cm long, nearly completely enveloped by 4 or 5 sterile, broadly ovate sheaths; sheath 1.5–1.8 cm long, scaled, light brownish; rachis about 6.5 cm long, with many flowers spirally arranged into a compact spike-like cylindrical raceme, opening simultaneously; floral bracts light brownish, dry papyraceous, oblong-lanceolate, ca. 6.5 mm long, almost twice as long as the pedicel and ovary, apex apiculate; pedicel and ovary ca. 3.5 mm, longitudinally slightly grooved. Flowers resupinate, not much opening, with unpleasant fishy smell. Sepals 3–6-veined, greenish-yellow, without spots or stripes. Dorsal sepal oblong-ovate or narrowly ovate, 4.5–5.0 mm × 1.0–1.5 mm, 3-veined, abaxially sparsely papillate, apex shortly aristate or apiculate, often recurved. Lateral sepals obliquely oblong-ovate, 5.5–6 mm × 2.0–2.5 mm, 5–6-veined, abaxially sparsely papillate, base adnate to column foot, margins strongly involute, with the apex shortly caudate, loosely adhering along the lower edges, forming an ovoid sac. Petals pale greenish-yellow, obliquely and broadly-based triangular, apex long acuminate, tapering into a long filiform or threadlike tail, usually contorted to spirally twisted, 2.0–2.2 mm × 0.8–1.0 mm near the base, with a tail 2.8–3.0 mm long, margins somewhat irregularly denticulate or erose. Lip yellowish-orange or deep golden yellow, fleshy, oblong narrowly ovoid, 2.5–2.8 mm × 1.3–1.5 mm, mobile on a thin ligament, recurved near the base, conspicuously auriculate at the base; disc with a longitudinal central groove, apex rounded or obtuse; auricles narrowly falcate, arching forward and gradually tapering to a long sharp point at the apex; densely glandular papillose on both sides and conspicuously ciliolate along margins, whitish. Column greenish-yellow, subcylindric, 2.0–2.2 mm tall; foot ca. 1.5 mm; stelidia well-developed and subulate, ca. 1.2 mm, often twisted inwards, equalling or slightly exceeding operculum, with a sharp point along the upper margin. Anther abaxially with a rounded crest, golden-yellow. Pollinia 4, the inner ones more than half as long as the outer ones.

**Figure 1.**
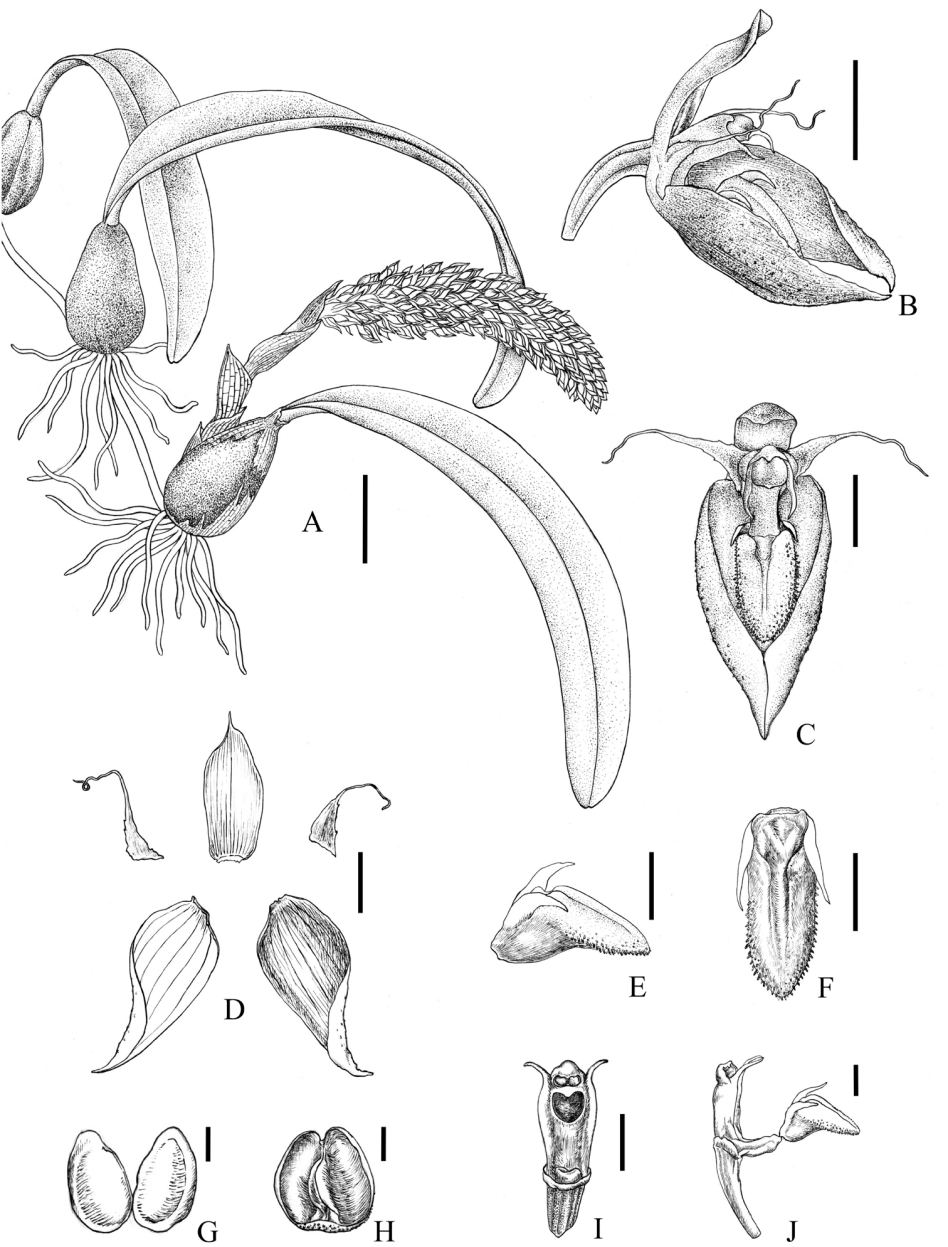
*Bulbophyllumchrysolabium.***A** Habit **B** Flower, lateral view **C** Flower, frontal view **D** Dorsal sepal, petals and lateral sepals, adaxial view **E** Lip, lateral view **F** Lip, ventral view **G** Pollinia **H** Operculum, ventral view **I** Column, ventral view **J** Column and lip, lateral view. Scale bars: 2 cm (**A**), 2 mm (**B–D**), 1 mm (**E–F, I–J**), 0.2 mm (**G–H**). Drawn by Yun-Xiao Liu.

#### Distribution and habitat.

So far known only from Menglian County in southwest Yunnan Province, China, growing as an epiphyte amongst mosses on the tree trunk near the edge of river in rather exposed circumstances in subtropical evergreen broad-leaved forest.

#### Etymology.

The specific epithet comes from the Ancient Greek word *chryso*- “golden” and the Latin derived *labium* “labellum”, referring to the golden-yellow lip of the type.

#### Phenology.

Flowering observed in December.

#### Conservation status.

*Bulbophyllumchrysolabium* is known only from its type locality, where it is represented by a single population of about 30–50 individual plants or clumps. The extent of occurrence (EOO) of the species cannot be estimated and its area of occupancy (AOO) is estimated to be 4 km^2^, which falls within the thresholds for Critically Endangered under criterion B2. Additional individuals of this species may yet be found in adjacent areas as the investigation continues. The location is relatively inaccessible to casual hikers and the potential for habitat destruction is limited, but it is not in a national reserve or other kinds of protected areas. Fortunately, a few individuals were brought into cultivation at SCBG. Although there is limited data to determine its actual distribution range, biology and threats, the current available information (one location in a non-protected area and AOO less than 10 km^2^) led us to assign a preliminary status of Critically Endangered [CR B2ab(iii)] according to the IUCN Red List Categories and Criteria (IUCN 2017).

**Figure 2.**
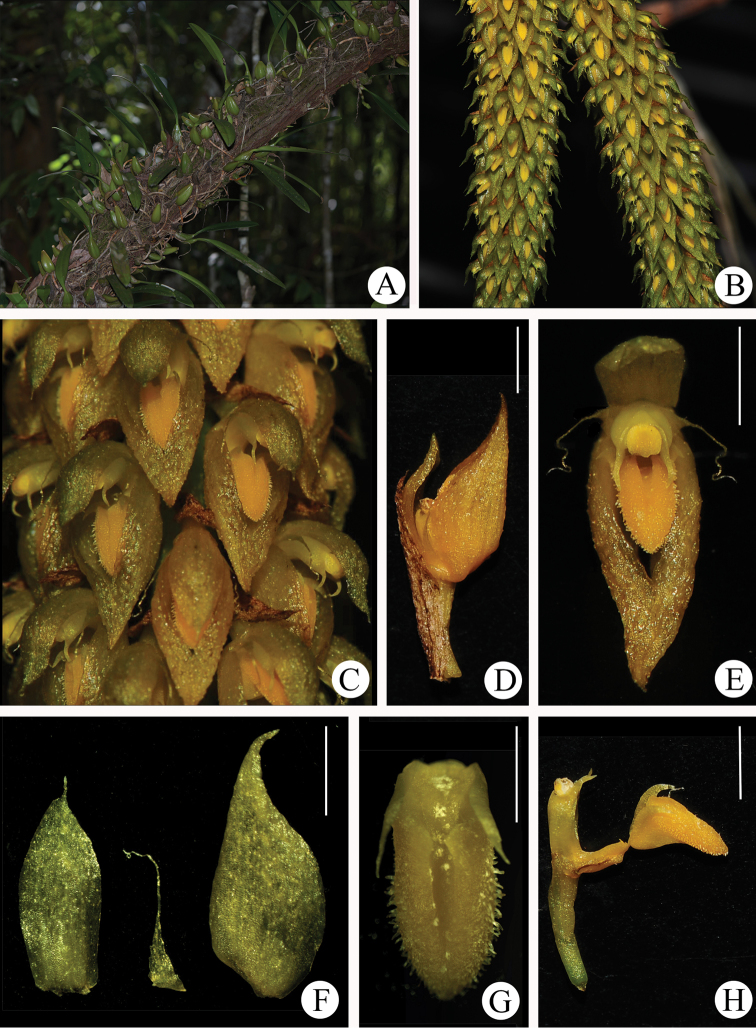
*Bulbophyllumchrysolabium.***A** Habitat **B** Inflorescences **C** Close-up of inflorescence **D** Flower, lateral view showing floral bract **E** Flower, frontal view **F** Dorsal sepal, petal and lateral sepal, abaxial view **G** Lip, ventral view **H** Column and lip, lateral view. Scale bars, 1 mm (**G**), 2 mm (**D–F, H**).

## Supplementary Material

XML Treatment for Bulbophyllum chrysolabium
